# Hydrogen induced redox mechanism in amorphous carbon resistive random access memory

**DOI:** 10.1186/1556-276X-9-52

**Published:** 2014-01-29

**Authors:** Yi-Jiun Chen, Hsin-Lu Chen, Tai-Fa Young, Ting-Chang Chang, Tsung-Ming Tsai, Kuan-Chang Chang, Rui Zhang, Kai-Huang Chen, Jen-Chung Lou, Tian-Jian Chu, Jung-Hui Chen, Ding-Hua Bao, Simon M Sze

**Affiliations:** 1 Department of Mechanical and Electro-Mechanical Engineering, National Sun Yat-Sen University, Kaohsiung 804, Taiwan; 2 Department of Physics, National Sun Yat-Sen University, Kaohsiung 804, Taiwan; 3 Department of Materials and Optoelectronic Science, National Sun Yat-Sen University, Kaohsiung 804, Taiwan; 4 School of Software and Microelectronics, Peking University, BeiJing 100871, People’s Republic of China; 5 Department of Electronics Engineering and Computer Science, Tung-Fang Design University, Kaohsiung 829, Taiwan; 6 Department of Chemistry, National Kaohsiung Normal University, Kaohsiung 802, Taiwan; 7 State Key Laboratory of Optoelectronic Materials and Technologies, School of Physics and Engineering, Sun Yat-Sen University, Guangzhou 510275, People’s Republic of China; 8 Department of Electrical Engineering, Stanford University, Stanford, CA 94305, USA

**Keywords:** Carbon, Hydrogen redox, Conjugation double bond, RRAM

## Abstract

We investigated the bipolar resistive switching characteristics of the resistive random access memory (RRAM) device with amorphous carbon layer. Applying a forming voltage, the amorphous carbon layer was carbonized to form a conjugation double bond conductive filament. We proposed a hydrogen redox model to clarify the resistive switch mechanism of high/low resistance states (HRS/LRS) in carbon RRAM. The electrical conduction mechanism of LRS is attributed to conductive sp^2^ carbon filament with conjugation double bonds by dehydrogenation, while the electrical conduction of HRS resulted from the formation of insulating sp^3^-type carbon filament through hydrogenation process.

## Background

Recently, portable electronic products which are combined memory circuits [1-3], display design [4,5] and IC circuits have popularized considerably in the last few years. To surmount the technical and physical limitation issues of conventional charge-storage-based memories [6-11], the resistance random access memory (RRAM) is constructed of an insulating layer sandwiched by two electrodes. This structure is a great potential candidate for next-generation nonvolatile memory due to its superior characteristics such as lesser cost, simple structure, high-speed operation, and nondestructive readout [12-21].

The carbon-based resistive memory (C-RRAM) has emerged as one of a few candidates with high density and low power. The resistive switching of C-RRAM relies on the formation and rupture of filaments due to redox chemical reaction mechanism, which is similar to most other reported RRAM devices [22-43].

In this paper, we investigated the resistive switching characteristics of amorphous carbon films prepared by RF magnetron sputter deposition technique for nonvolatile memory applications. Reliable and reproducible switching phenomena of the amorphous carbon RRAM with Pt/a-C:H/TiN structure were observed. In addition, the resistive switching mechanism of the amorphous carbon RRAM device is discussed and verified by electrical and material analysis.

## Methods

The experimental specimens were prepared as follows. The carbon thin film (around 23 nm) was deposited on the TiN/Ti/SiO_2_/Si substrate by RF magnetron sputtering with a carbon target. After that, the Pt top electrode of 200-nm thickness was deposited on the specimen by DC magnetron sputtering. The photolithography and lift-off technique were used to shape the cells into square pattern with area of 0.36 to 16 μm^2^. The electrical measurements of devices were performed using Agilent B1500 semiconductor parameter analyzer (Santa Clara, CA, USA). Besides, Fourier transform infrared spectroscopy (FTIR) and Raman spectroscopy were used to analyze the chemical composition and bonding of the amorphous carbon materials, respectively.

## Results and discussion

**Figure 1:**
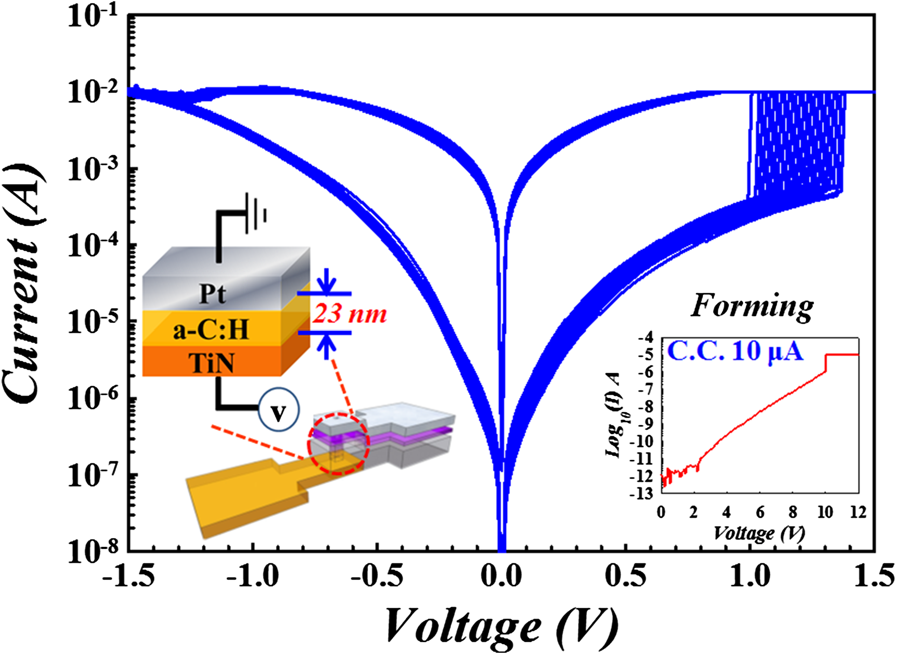
Current–voltage sweeps of Pt/a-C:H/TiN memory device.

Figure 1 shows the bipolar current–voltage (I-V) characteristics of the carbon memory cell in semi-logarithmic scale under DC voltage sweeping mode at room temperature. After the electroforming process (inset of Figure 1), the resistance switching behavior of the as-fabricated device can be obtained repeatedly, using DC voltage switching with a compliance current of 10 μA. By sweeping the bias from zero to negative value (about -1.5 V), the resistance state is transformed from low resistance states (LRS) to high resistance states (HRS), called as ‘reset process’. Conversely, as the voltage sweeps from zero to a positive value (about 1.5 V), the resistance state is turned back to LRS, called as ‘set process’. During set process, a compliance current of 10 mA is applied to prevent permanent breakdown.

**Figure 2:**
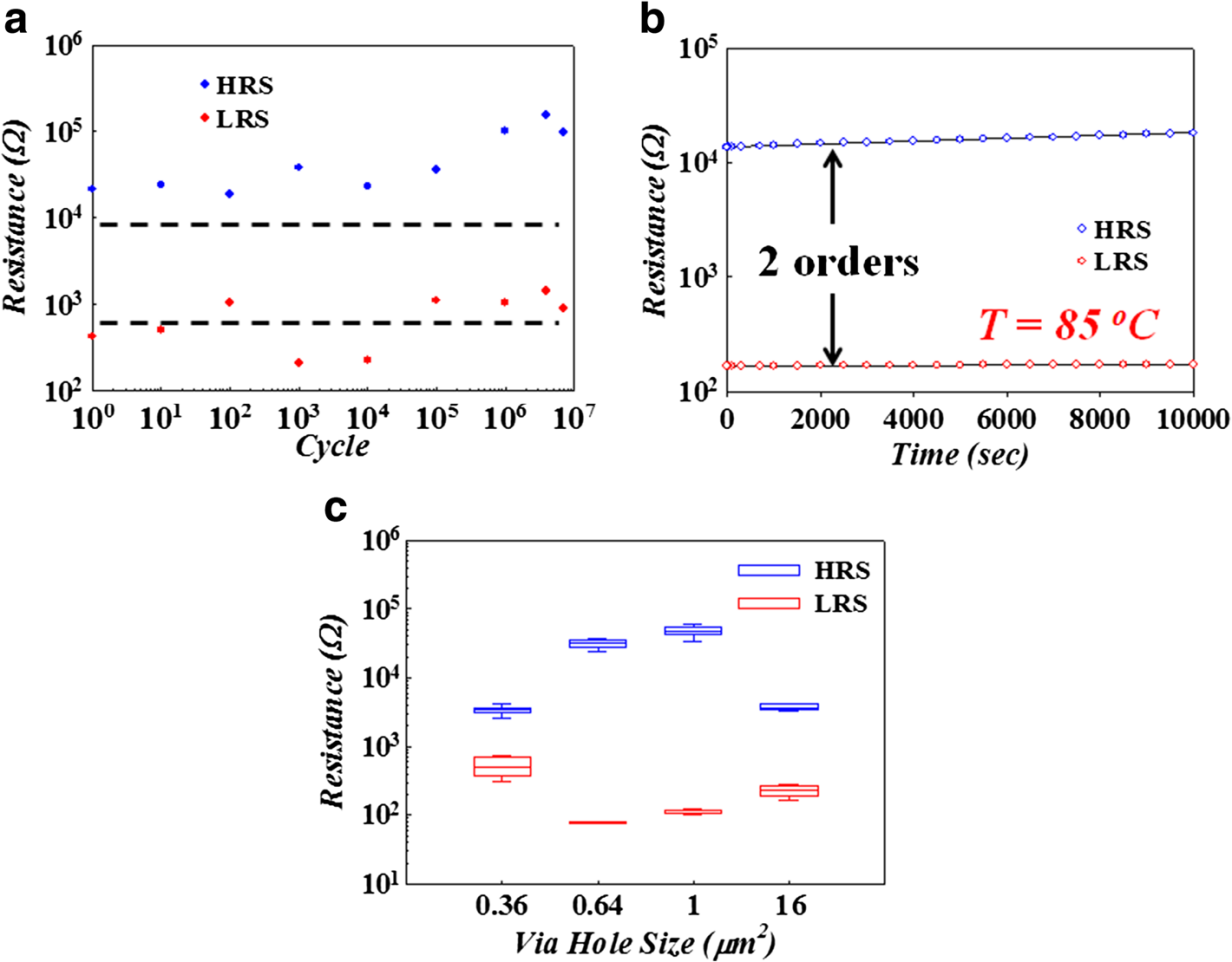
Endurance (a), retention properties (b), and sizing effect measurement (c) of Pt/a-C:H/TiN memory device.

To further evaluate the memory performance of amorphous carbon RRAM, the endurance and retention tests were shown in Figure 2. The resistance values of reliability and sizing effect measurement were obtained by a read voltage of 0.2 V. The device exhibits stable HRS and LRS even after more than 10^7^ sweeping cycles (Figure 2a), which demonstrates its acceptable switching endurance capability. The retention characteristics of HRS and LRS at *T* = 85°C are shown in Figure 2b. No significant degradation of resistance in HRS and LRS was observed. It indicates that the device has good reliability for nonvolatile memory applications. Figure 2c reveals the resistance of LRS and HRS states with various sizes of via hole, which is independent with the electrode area of the device. According to the proposed model by Sawa [44], the resistive switching behavior in carbon RRAM is attributed to filament-type RRAM.

**Figure 3:**
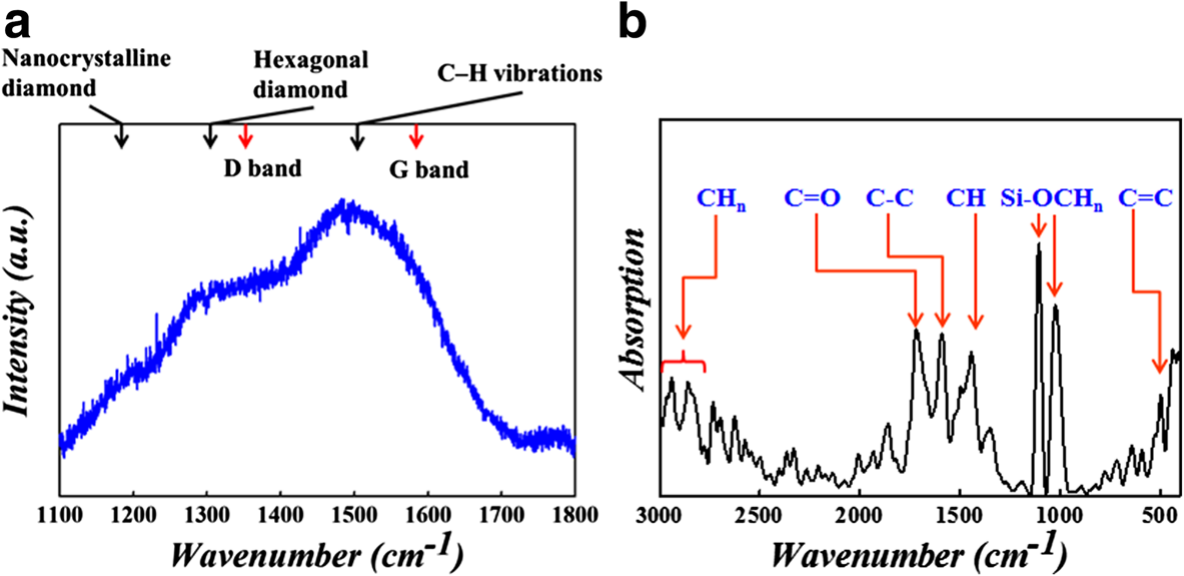
**Raman spectra of C sp**^**2 **^**and C sp**^**3 **^**in amorphous carbon film (a); FTIR spectrum of amorphous carbon film (b).**

To investigate the interesting phenomena, we utilized the material spectrum analyses to find out the reason of working current reduction and better stability. The sputtered carbon film was analyzed by Raman spectroscopy and the spectra revealed in Figure 3a. The broaden peak from 1,100 to 1,700 cm^-1^ demonstrates the existence of amorphous carbon structure [45].

In order to further testify the existence of the carbon layer and find its chemical bonding type, FTIR was used to analyze the sputtered carbon thin film. C-H stretch peak can be observed at the wave number of 2,800 to 3,000 cm^-1^, as shown in the FTIR spectra of Figure 3b.

**Figure 4:**
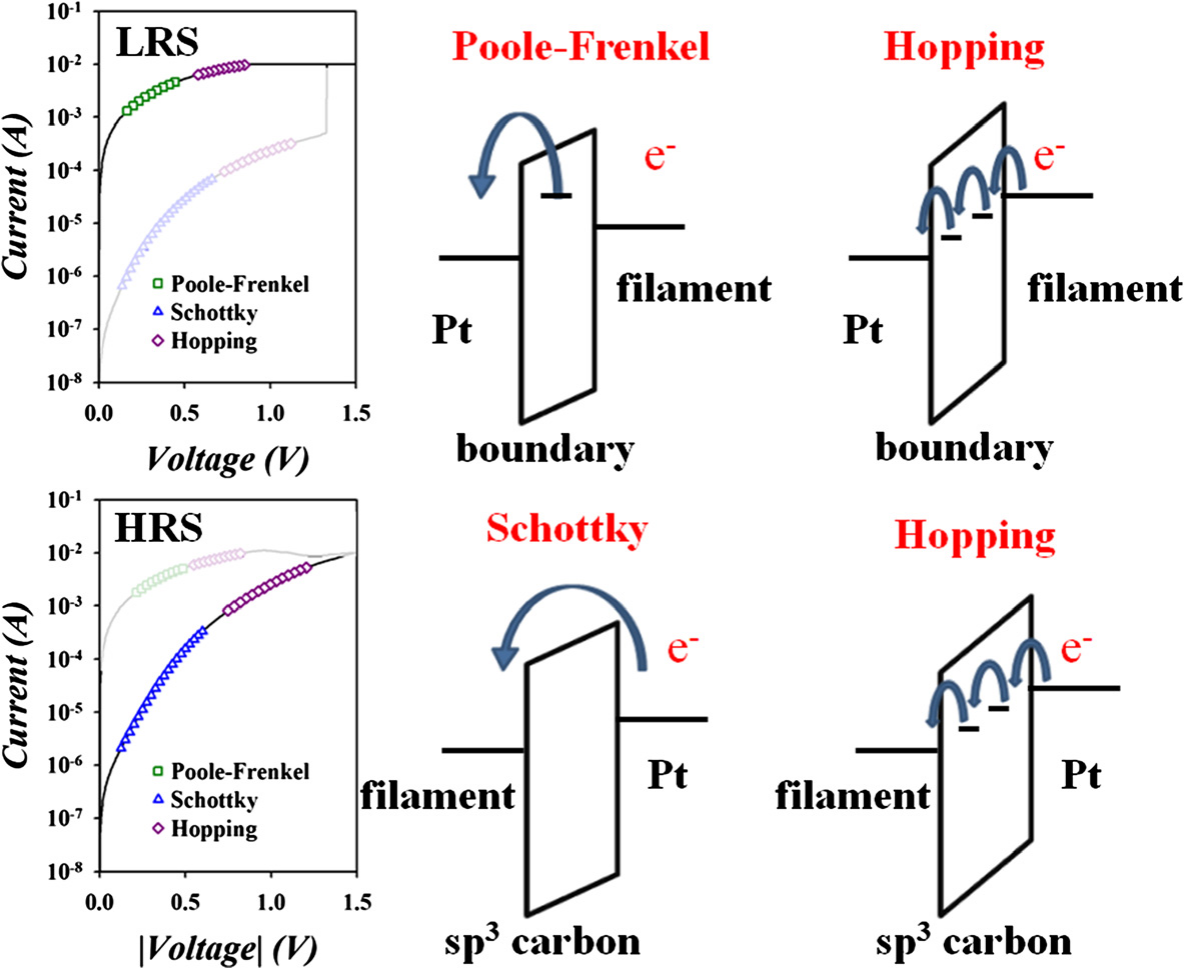
I-V curve fitting of Pt/a-C:H/TiN memory device with various carrier transport mechanisms.

To clarify the current transportation mechanism, the current vs. voltage (I-V) is presented in Figure 4. The LRS shows symmetric I-V curve at positive and negative electrical field. The electron transport exhibits Poole-Frenkel and Hopping conduction at middle and high voltage. However, the I-V curve is asymmetric in HRS, but the current transportation mechanism is Schottky emission and Hopping at middle and high voltage. The resistive switching mechanism of LRS and HRS is given in detail as follows.

**Figure 5:**
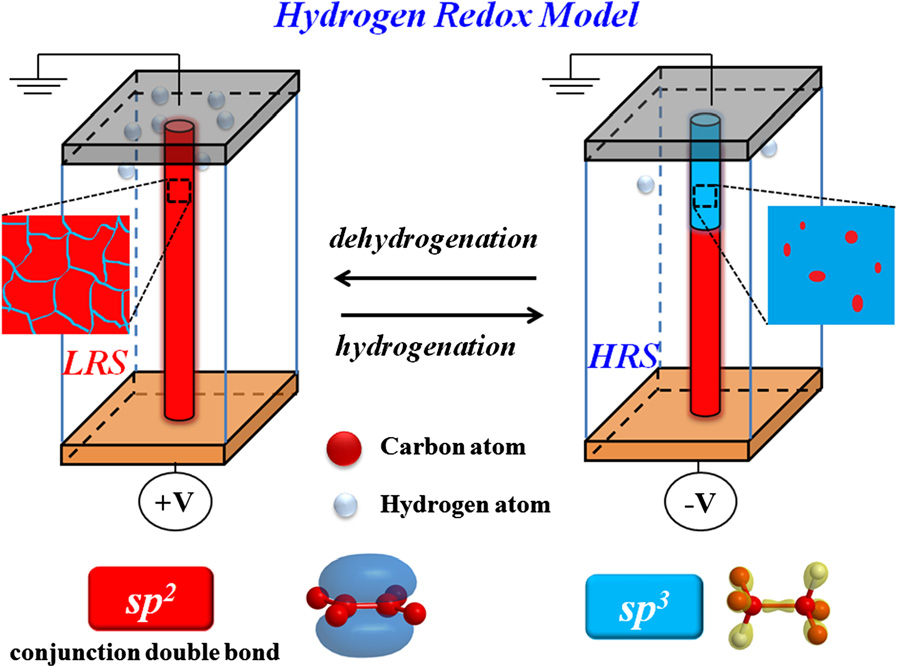
Hydrogen redox model of Pt/a-C:H/TiN memory device in LRS and HRS states.

On the basis of the electrical and material analyses, we proposed a reaction model to explain the transfer of carrier conduction mechanism of the amorphous carbon RRAM as shown in Figure 5. The conductive filament will be formed after the forming process, which is attributed to the connection between sp^2^ carbon fractions in the amorphous carbon layer [46]. Due to the current compliance, there is remaining amorphous carbon between conductive sp^2^ regions, as shown in left insert of Figure 5. Because the current pass through the boundaries of sp^2^ regions, the current fitting is dominated by Poole-Frenkel conduction in LRS. As higher voltage was applied, the significant barrier lowering caused the conduction dominated by hopping conduction through conjugation double bonds of sp^2^ carbon filament. When the bottom TiN electrode is applied with a negative bias to perform a reset process, hydrogen atoms were pulled from the Pt electrode and absorbed by double bonds of sp^2^ carbon, namely hydrogenation process. The hydrogenation reaction will transfer the conductive sp^2^ carbon filament into insulated sp^3^ carbon filament. As shown in the right insert of Figure 5, the region of filament near Pt electrode forms insulated sp^3^ carbon dominated, which leads to the current conduction exhibit Schottky conduction in HRS. The Hopping conduction is attributed to significant barrier lowering as the higher voltage was applied. Contrariwise, the hydrogen atoms were repelled to Pt electrode to form sp^2^ carbon filament during set process, called as dehydration process. Based on the hydrogen redox model, a repeatable switching behavior can be obtained in C-RRAM device.

## Conclusion

In conclusion, the amorphous carbon RRAM has been fabricated to investigate the resistive switching characteristics. The device has good resistive switching properties due to hydrogenation and dehydrogenation of H atoms in carbon RRAM. The material and electrical analyses give convincing evidence of hydrogen redox induced resistance switching in amorphous carbon RRAM. The current conduction of LRS was contributed to formation of conjugation double bonds in the carbon layer after dehydrogenation. Moreover, the current conduction of HRS was dominated by insulating sp^3^ carbon after hydrogenation at a reverse electrical filed.

## Competing interests

The authors declare that they have no competing interests.

## Authors’ contributions

YJC designed and set up the experimental procedure. HLC conducted the electrical measurement of the devices. TCC and TFY planned the experiments and agreed with the paper’s publication. TMT, KCC, KHC, and JCL revised manuscript critically and make some changes. RZ fabricated the devices with the assistance of TJC. JHC performed the Raman and FTIR spectra measurement. DHB and SMS assisted in the data analysis. All authors read and approved the final manuscript.

## References

[B1] Guan WH Long SB Jia R Liu M Nonvolatile resistive switching memory utilizing gold nanocrystals embedded in zirconium oxide Appl Phys Lett 2007 9 062111 10.1063/1.2760156

[B2] Liu Q Guan WH Long SB Jia R Liu M Chen JN Resistive switching memory effect of ZrO_2_ films with Zr^+^ implanted Appl Phys Lett 2008 9 012117 10.1063/1.2832660

[B3] Chang TC Jian FY Chen SC Tsai YT Developments in nanocrystal memory Mater Today 2011 9 608 615 10.1016/S1369-7021(11)70302-9

[B4] Tsai CT Chang TC Chen SC Lo IK Tsao SW Hung MC Chang JJ Wu CY Huang CY Influence of positive bias stress on N_2_O plasma improved InGaZnO thin film transistor Appl Phys Lett 2010 9 242105 10.1063/1.3453870

[B5] Chen TC Chang TC Tsai CT Hsieh TY Chen SC Lin CS Hung MC Tu CH Chang JJ Chen PL Behaviors of InGaZnO thin film transistor under illuminated positive gate-bias stress Appl Phys Lett 2010 9 112104 10.1063/1.3481676

[B6] Liu J Wang Q Long SB Zhang MH Liu M A metal/Al_2_O_3_/ZrO_2_/SiO_2_/Si (MAZOS) structure for high-performance non-volatile memory application Semicond Sci Technol 2010 9 055013 10.1088/0268-1242/25/5/055013

[B7] Jiang DD Zhang MH Huo ZL Wang Q Liu J Yu ZA Yang XN Wang Y Zhang B Chen JN Liu M A study of cycling induced degradation mechanisms in Si nanocrystal memory devices Nanotechnology 2011 9 254009 10.1088/0957-4484/22/25/254009 21572215

[B8] Syu YE Chang TC Tsai TM Hung YC Chang KC Tsai MJ Kao MJ Sze SM Redox reaction switching mechanism in RRAM device with Pt/CoSiO_X_/TiN structure IEEE Electron Device Lett 2011 9 545 547

[B9] Chen MC Chang TC Tsai CT Huang SY Chen SC Hu CW Sze SM Tsai MJ Influence of electrode material on the resistive memory switching property of indium gallium zinc oxide thin films Appl Phys Lett 2010 9 262110 10.1063/1.3456379

[B10] Zhu CX Huo ZL Xu ZG Zhang MH Wang Q Liu J Long SB Liu M Performance enhancement of multilevel cell nonvolatile memory by using a bandgap engineered high-κ trapping layer Appl Phys Lett 2010 9 253503 10.1063/1.3531559

[B11] Zhu CX Xu ZG Huo ZL Yang R Zheng ZW Cui YX Liu J Wang YM Shi DX Zhang GY Li FH Liu M Investigation on interface related charge trap and loss characteristics of high-k based trapping structures by electrostatic force microscopy Appl Phys Lett 2011 9 223504 10.1063/1.3664222

[B12] Tsai TM Chang KC Chang TC Syu YE Chuang SL Chang GW Liu GR Chen MC Huang HC Liu SK Tai YH Gan DS Yang YL Young TF Tseng BH Chen KH Tsai MJ Ye C Wang H Sze SM Bipolar resistive RAM characteristics induced by nickel incorporated into silicon oxide dielectrics for IC applications IEEE Electron Device Lett 2012 9 1696 1698

[B13] Fu D Xie D Feng TT Zhang CH Niu JB Qian H Liu LT Unipolar resistive switching properties of diamondlike carbon-based RRAM devices IEEE Electron Device Lett 2011 9 803 805

[B14] Zhuge F Dai W He CL Wang AY Liu YW Li M Wu YH Cui P Li RW Nonvolatile resistive switching memory based on amorphous carbon Appl Phys Lett 2010 9 163505 10.1063/1.3406121

[B15] Peng PG Xie D Yang Y Zhou CJ Ma S Feng TT Tian H Ren TL Bipolar and unipolar resistive switching effects in an Al/DLC/W structure J Phys D Appl Phys 2012 9 365103 10.1088/0022-3727/45/36/365103

[B16] Rueckes T Kim K Joselevich E Tseng GY Cheung CL Lieber CM Carbon nanotube-based nonvolatile random access memory for molecular computing Science 2000 9 94 97 10.1126/science.289.5476.94 10884232

[B17] Wang Y Liu Q Long SB Wang W Wang Q Zhang MH Zhang S Li YT Zuo QY Yang JH Liu M Investigation of resistive switching in Cu-doped HfO_2_ thin film for multilevel non-volatile memory applications Nanotechnology 2010 9 045202 10.1088/0957-4484/21/4/045202 20009169

[B18] Kuang YB Huang R Ding W Zhang LJ Wang YG Flexible single-component-polymer resistive memory for ultrafast and highly compatible nonvolatile memory applications IEEE Electron Device Lett 2010 9 758 760

[B19] Russo U Ielmini D Cagli C Lacaita AL Filament conduction and reset mechanism in NiO-Based Resistive-Switching Memory (RRAM) Devices IEEE Trans Electron Devices 2009 9 186 192

[B20] Standley B Bao WZ Zhang H Bruck J Lau CN Bockrath M Graphene-based atomic-scale switches Nano Lett 2008 9 3345 3349 10.1021/nl801774a 18729415

[B21] Li YT Long SB Zhang MH Liu Q Zhang S Wang Y Zuo QY Liu S Liu M Resistive switching properties of Au/ZrO_2_/Ag structure for low-voltage nonvolatile memory applications IEEE Electron Device Lett 2010 9 117 119

[B22] Sebastian A Pauza A Rossel C Shelby RM Rodríguez AF Pozidis H Eleftheriou E Resistance switching at the nanometre scale in amorphous carbon New J Phys 2011 9 013020 10.1088/1367-2630/13/1/013020

[B23] Chang KC Tsai TM Zhang R Chang TC Chen KH Chen JH Young TF Lou JC Chu TJ Shih CC Pan JH Su YT Syu YE Tung CW Chen MC Wu JJ Hu Y Sze SM Electrical conduction mechanism of Zn:SiO_x_ resistance random access memory with supercritical CO_2_ fluid process Appl Phys Lett 2013 9 083509 10.1063/1.4819162

[B24] Chang KC Zhang R Chang TC Tsai TM Lou JC Chen JH Young TF Chen MC Yang YL Pan YC Chang GW Chu TJ Shih CC Chen JY Pan CH Su YT Syu YE Tai YH Sze SM Origin of hopping conduction in graphene-oxide-doped silicon oxide resistance random access memory devices IEEE Electron Device Lett 2013 9 677 679

[B25] Zhang R Chang KC Chang TC Tsai TM Chen KH Lou JC Chen JH Young TF Shih CC Yang YL Pan YC Chu TJ Huang SY Pan CH Su YT Syu YE Sze SM High performance of graphene oxide-doped silicon oxide-based resistance random access memory Nanoscale Res Lett 2013 9 497 10.1186/1556-276X-8-497 24261454 PMC3874615

[B26] Tsai TM Chang KC Zhang R Chang TC Lou JC Chen JH Young TF Tseng BH Shih CC Pan YC Chen MC Pan JH Syu YE Sze SM Performance and characteristics of double layer porous silicon oxide resistance random access memory Appl Phys Lett 2013 9 253509 10.1063/1.4812474

[B27] Chang KC Pan CH Chang TC Tsai TM Zhang R Lou JC Young TF Chen JH Shih CC Chu TJ Chen JY Su YT Jiang JP Chen KH Huang HC Syu YE Gan DS Sze SM Hopping effect of hydrogen-doped silicon oxide insert RRAM by supercritical CO_2_ fluid treatment IEEE Electron Device Lett 2013 9 617 619

[B28] Chang KC Tsai TM Chang TC Wu HH Chen KH Chen JH Young TF Chu TJ Chen JY Pan CH Su YT Syu YE Tung CW Chang GW Chen MC Huang HC Tai YH Gan DS Wu JJ Hu Y Sze SM Low temperature improvement method on Zn:SiO_x_ resistive random access memory devices IEEE Electron Device Lett 2013 9 511 513

[B29] Chang KC Tsai TM Chang TC Wu HH Chen JH Syu YE Chang GW Chu TJ Liu GR Su YT Chen MC Pan JH Chen JY Tung CW Huang HC Tai YH Gan DS Sze SM Characteristics and mechanisms of silicon-oxide-based resistance random access memory IEEE Electron Device Lett 2013 9 399 401

[B30] Tsai TM Chang KC Chang TC Chang GW Syu YE Su YT Liu GR Liao KH Chen MC Huang HC Tai YH Gan DS Ye C Wang H Sze SM Origin of hopping conduction in Sn-doped silicon oxide RRAM with supercritical CO_2_ fluid treatment IEEE Electron Device Lett 2012 9 1693 1695

[B31] Tsai TM Chang KC Chang TC Syu YE Liao KH Tseng BH Sze SM Dehydroxyl effect of Sn-doped silicon oxide resistance random access memory with supercritical CO_2_ fluid treatment Appl Phys Lett 2012 9 112906 10.1063/1.4750235

[B32] Chang KC Huang JW Chang TC Tsai TM Chen KH Young TF Chen JH Zhang R Lou JC Huang SY Pan YC Huang HC Syu YE Gan DS Bao DH Sze SM Space electric field concentrated effect for Zr:SiO_2_ RRAM devices using porous SiO_2_ buffer layer Nanoscale Res Lett 2013 9 523 10.1186/1556-276X-8-523 24330524 PMC3881491

[B33] Chang KC Tsai TM Chang TC Syu YE Chuang SL Li CH Gan DS Sze SM The effect of silicon oxide based RRAM with tin doping Electrochem Solid-State Lett 2012 9 H65 H68 10.1149/2.013203esl

[B34] Chang KC Tsai TM Chang TC Syu YE Wang CC Chuang SL Li CH Gan DS Sze SM Reducing operation current of Ni-doped silicon oxide resistance random access memory by supercritical CO_2_ fluid treatment Appl Phys Lett 2011 9 263501 10.1063/1.3671991

[B35] Syu YE Chang TC Tsai TM Chang GW Chang KC Lou JH Tai YH Tsai MJ Wang YL Sze SM Asymmetric carrier conduction mechanism by tip electric field in WSiO_X_ resistance switching device IEEE Electron Device Lett 2012 9 3 342 344

[B36] Long SB Perniola L Cagli C Buckley J Lian XJ Miranda E Pan F Liu M Sune J Voltage and power-controlled regimes in the progressive uni-polar RESET transition of HfO_2_-based RRAM Sci Rep 2013 9 2929 24121547 10.1038/srep02929 PMC3796310

[B37] Syu YE Chang TC Lou JH Tsai TM Chang KC Tsai MJ Wang YL Liu M Sze SM Atomic-level quantized reaction of HfO_x_ memristor Appl Phys Lett 2013 9 172903 10.1063/1.4802821

[B38] Long SB Lian XJ Cagli C Perniola L Miranda E Liu M Sune J A model for the set statistics of RRAM inspired in the percolation model of oxide breakdown IEEE Electron Device Lett 2013 9 8 999 1001

[B39] Chu TJ Chang TC Tsai TM Wu HH Chen JH Chang KC Young TF Chen KH Syu YE Chang GW Chang YF Chen MC Lou JH Pan JH Chen JY Tai YH Ye C Wang H Sze SM Charge quantity influence on resistance switching characteristic during forming process IEEE Electron Device Lett 2013 9 4 502 504

[B40] Long SB Lian XJ Cagli C Cartoixa X Rurali R Miranda E Jimenez D Perniola L Liu M Sune J Quantum-size effects in hafnium-oxide resistive switching Appl Phys Lett 2013 9 18 183505 10.1063/1.4802265

[B41] Su YT Chang KC Chang TC Tsai TM Zhang R Lou JC Chen JH Young TF Chen KH Tseng BH Shih CC Yang YL Chen MC Chu TJ Pan CH Syu YE Sze SM Characteristics of hafnium oxide resistance random access memory with different setting compliance current Appl Phys Lett 2013 9 16 163502 10.1063/1.4825104

[B42] Zhang R Tsai TM Chang TC Chang KC Chen KH Lou JC Young TF Chen JH Huang SY Chen MC Shih CC Chen HL Pan JH Tung CW Syu YE Sze SM Mechanism of power consumption inhibitive multi-layer Zn:SiO_2_/SiO_2_ structure resistance random access memory J 2013 9 234501 10.1063/1.4843695

[B43] Chang KC Chen JH Tsai TM Chang TC Huang SY Zhang R Chen KH Syu YE Chang GW Chu TJ Liu GR Su YT Chen MC Pan JH Liao KH Tai YH Young TF Sze SM Ai CF Wang MC Huang JW Improvement mechanism of resistance random access memory with supercritical CO_2_ fluid treatment J. of Supercritical Fluids 2014 9 183 189

[B44] Sawa A Resistive switching in transition metal oxides Mater Today 2008 9 28 36

[B45] Schwan J Ulrich S Batori V Ehrhardt H Silva SRP Raman spectroscopy on amorphous carbon films J Appl Phys 1996 9 440 447 10.1063/1.362745

[B46] Evtukh A Litovchenko V Semenenko M Yilmazoglu O Mutamba K Hartnagel HL Pavlidis D Formation of conducting nanochannels in diamond-like carbon films Semicond Sci Technol 2006 9 1326 1330 10.1088/0268-1242/21/9/018

